# *In vitro* evaluation of probiotic strains for lactic acid production

**DOI:** 10.4317/jced.55232

**Published:** 2019-04-01

**Authors:** Ahmed Faraz, Rao Arathi, Saralaya Vishwas, Natarajan Srikanth, Yarmunja-Mahabala Karuna, Baranya-Srikrishna Suprabha

**Affiliations:** 1 Ex Post Graduate Student. Department of Paedodontics and Preventive Dentistry Manipal College of Dental Sciences, Mangalore, Manipal Academy of Higher Education, Manipal; 2 Professor. Department of Paedodontics and Preventive Dentistry Manipal College of Dental Sciences, Mangalore, Manipal Academy of Higher Education, Manipal; 3 Associate Professor. Department of Microbiology Kasturba Medical College, Mangalore, Manipal Academy of Higher Education, Manipal; 4 Professor. Department of Oral Pathology Manipal College of Dental Sciences, Mangalore, Manipal Academy of Higher Education, Manipal; 5 Assistant Professor. Department of Paedodontics and Preventive Dentistry Manipal College of Dental Sciences, Mangalore, Manipal Academy of Higher Education, Manipal; 6 Professor & Head. Department of Paedodontics and Preventive Dentistry Manipal College of Dental Sciences, Mangalore, Manipal Academy of Higher Education, Manipal

## Abstract

**Background:**

The growing interest on usage of probiotic lactobacilli in maintaining oral health has posed number of questions on its probable side effects. One such consideration could be an increased acid production in dental plaque, in turn leading to dental caries. Thus, the aim of this study was to comparatively evaluate the lactic acid producing ability of *L. acidophilus* and *L. plantarum* with and without dental plaque.

**Material and Methods:**

The study consisted of five groups: 3 control groups (*Supragingival plaque, L. acidophilus* and *L. plantarum*) and 2 test groups (Supragingival plaque with *L. acidophilu*s and Supragingival plaque with *L. plantarum*). 26 samples for each group were collected and their baseline spectrophotometric values were recorded. The acid production was initiated by adding 25?l fructose (10%) and stopped by centrifugation for 2 min. The concentration of the lactic acid produced was determined with the aid of COBAS INTEGRA 400 plus.

**Results:**

On comparison of Lactic Acid estimation in mg/dl, the mean values of Plaque group was the highest followed by Plaque +L acidophilus, Plaque +L plantarum, L acidophilus and least in L plantarum. The posthoc analysis shows that the comparison of Group 1 (Plaque) and Group 2 (Plaque +L acidophilus) is statistically Significant results between all the groups except between the Plaque +L acidophilus and Plaque +L plantarum group.

**Conclusions:**

The lactic acid producing ability of pure suspensions of *L.acidophilus* and *L.plantarum* and the lactic acid producing efficiency becomes more when they are added to the supragingival plaque.

** Key words:**Probiotic, lactic Acid, plaque.

## Introduction

Dental caries is the most prevailing infectious disease worldwide which results due to the prolonged interaction between microbes and fermentable carbohydrates (1). Thus, any preventive measures targeted at the cariogenic microorganisms and/or fermentable sugars is found to be beneficial (2). Thus, antimicrobial approach aids to control the caries process, especially in those who are at high risk for caries (3). One such antimicrobial approach which is currently gaining the popularity is the use of Probiotics to counteract caries.

Probiotics are living microorganisms that are risk free for human consumption and, when ingested in adequate amounts, have a positive influence on human wellbeing, nourishment and health (4). The most commonly used probiotic strains are found in the oral cavity and they belong to the genera Lactobacillus and Bifidobacterium. Theoretically these are known to cause dental caries as they are excellent acidogenic and aciduric microorganisms. Additionally, these microorganisms are frequently seen in carious lesions. However, according to the existing evidence probiotics decrease the caries risk in a caries prone individual (5).

Though lactobacillus under normal conditions produces lactic acid as an end product of fermentation process, when it comes in contact with the plaque, it reduces the amount of lactic acid production thus reducing the cariogenesity. It was found that the various species of *Lactobacillus* including *L. paracasei, L. plantarum*, and *L. rhamnosus* can interfere against *S. mutans* (6). The consumption of *L. rhamnosus GG, L. reuteri*, and *Bifidobacterium lactis* for a short period reduces *S. mutans* count as well as the dental plaque (7,8).

Among the lactobacillus species *Lactobacillus acidophilus* is known for its probiotic potential and its acid resistance. It is said to be substantially utilized as a subordinate for the anticipation of dental caries (6). Thus the present study was conducted to comparatively evaluate the lactic acid producing ability of two probiotic strains of *lactobacillus namely, L. acidophilus* and *L. plantarum* with and without dental plaque.

## Material and Methods

The study was initiated after obtaining Institutional Ethics Committee approval. Procedures followed in the study were in accordance with the Helsinki declaration of 1975 that was revised in 2000. Parents/guardians of the study subjects were given the complete details of the study and informed consent was obtained. Informed assent was taken from the children included in the study.

Sample size: By keeping the test power at 0.80 and predetermined Type I error of 0.5 as per cumulative distribution function (Cohen’s d- 0.8), the total sample size calculated was 130.

Inclusion and exclusion criteria: 130 children between the age of 7-12 years having > 3 decayed teeth were selected for the study. The children who were exposed to food items supplemented with probiotic bacteria, xylitol or any other antimicrobial agents and systemic antibiotics were excluded from the study.

-Study groups:

The 130 samples thus selected were randomly divided into five groups: Group 1- Supragingival plaque (Control Group), Group 2- Supragingival plaque with *Lactobacillus acidophilus*, Group 3- Supragingival plaque with *Lactobacillus plantarum*, Group 4- *Lactobacillus acidophilus* (Control Group) and Group 5- *Lactobacillus plantarum* (Control Group).

-Procedure.

Children were instructed not to brush their teeth for 24 hours before obtaining plaque samples. Supragingival plaque was collected using a sterile toothpick from all the teeth, which was transferred to a sterile test tube and diluted in 1:10 saline. The fresh samples were suspended and homogenized in PBS adjusted at pH=7.2 and to an optical density of OD=0.2 at 340 nm. Under anaerobic conditions, *Lactobacillus acidophilus* and *Lactobacillus plantarum* were cultivated in Man Rogosa Sharpe (MRS) broth at 37°C for 24 hours and then washed twice using Phosphate buffered saline (PBS).

One ml of the plaque suspension with no addition of probiotic bacteria served as control. Also, one ml suspensions of *Lactobacillus acidophilus* and *Lactobacillus plantarum* each with the same optical density were used as controls. Five hundred μl of the plaque suspensions were mixed with 500 μl of equally dense suspensions of *L. acidophilus* and *L. plantarum* in PBS to obtain group 2 and group 3 samples respectively.

Baseline spectrophotometric values were recorded for all the groups and then for 1 hour the suspensions were incubated at 37°C without agitation. The optical density (OD) readings were repeated and in each group the production of lactic acid was initiated by adding 25 μl fructose (10%). After 30 minutes of further incubation, centrifugation for 2 min (10,000 rpm) was done to stop the fermentation and supernatant was withdrawn for further analyses. Lactic acid (LA) concentration was determined with the aid of COBAS INTEGRA 400 plus (Central Labs, Bangalore, India).

-Statistical analysis

Data was processed using IBM-SPSS version 16 software. KOLMOGOROV SMIRNOV TEST shows that the variables are not skewed grossly. (as the values are not significant) ( Table 1). Thus parametric tests can be done. The test that would be used is one way anova with posthoc tukey test.

**Table 1:**
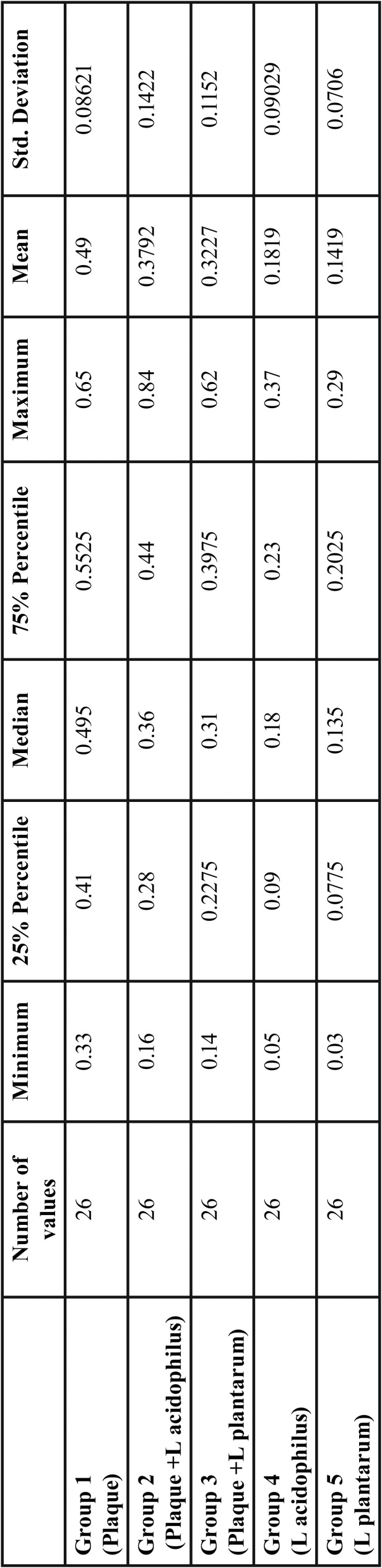
Descriptive Analysis of the Amount of lactic acid produced from different groups.

## Results

**Figure 1:**
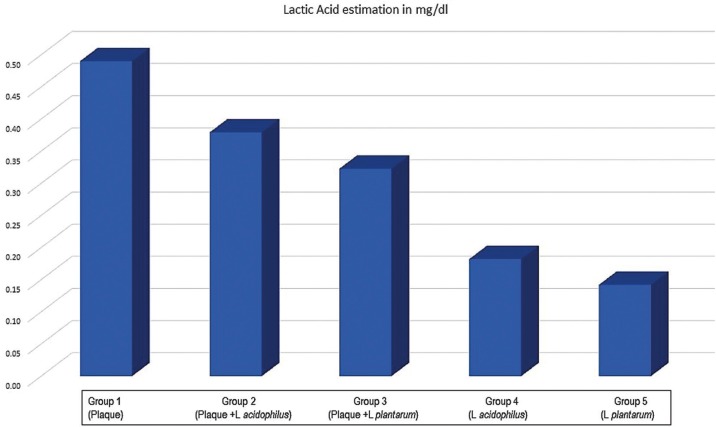
Mean values of Lactic Acid produced from different groups.

Mean values of lactic acid produced from different groups is given in Figure 1. On comparison of Lactic Acid estimation in mg/dl between the five groups the mean values of Group 1 (Plaque) was the highest followed by Group 2 (Plaque +L acidophilus), Group 3 (Plaque +L plantarum), Group 4 (L acidophilus) and least in Group 5(L plantarum) ( Table 2).

**Table 2:**
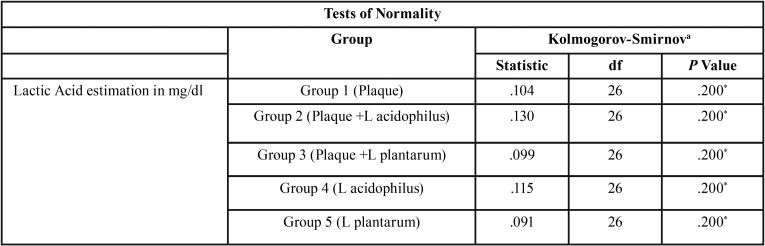
Test of Normality.

This comparison is significant with a statistics of 49.175 and *p* value of <0.001 ( Table 3). The posthoc analysis ( Table 4) shows that the comparison of Group 1 (Plaque) and Group 2 (Plaque +L acidophilus) is statistically Significant with a *p* value of 0.002. The comparison of Group 1 (Plaque) and Group 3 (Plaque +L plantarum) is statistically significant with a *p* value of <0.001. The comparison of Group 1 (Plaque) and Group 4 (L acidophilus) is statistically Significant with a *p* value of <0.001. The comparison of Group 1 (Plaque) and Group 5 (L plantarum) is statistically Significant with a *p* value of <0.001. The comparison of Group 2 (Plaque +L acidophilus) and Group 3 (Plaque +L plantarum) is statistically Not Significant with a *p* value of 0.291. The comparison of Group 2 (Plaque +L acidophilus) and Group 4 (L acidophilus) is statistically Significant with a *p* value of <0.001. The comparison of Group 2 (Plaque +L acidophilus) and Group 5c (L plantarum) is statistically Significant with a *p* value of <0.001. The comparison of Group 3 (Plaque +L plantarum) and Group 4 (L acidophilus) is statistically Significant with a *p* value of <0.001.The comparison of Group 3 (Plaque +L plantarum) and Group 5c (L plantarum) is statistically Significant with a *p* value of <0.001. The comparison of Group 4 (L acidophilus) and Group 5c (L plantarum) is statistically Not Significant with a *p* value of 0.637.

**Table 3:**
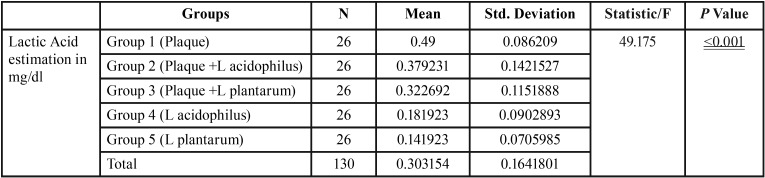
One way anova with posthoc tukey test.

**Table 4:**
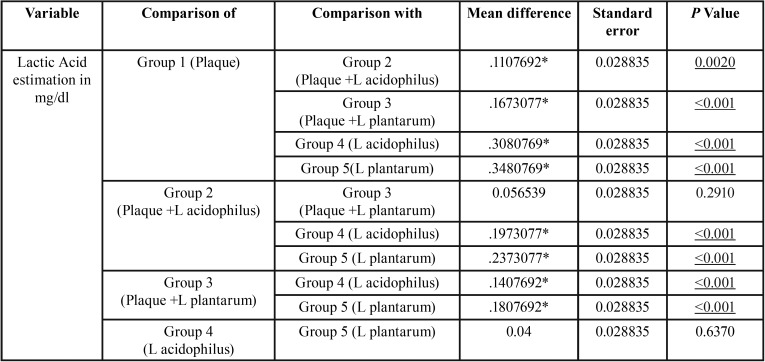
Posthoc tukey test.

## Discussion

To improve oral health by using probiotic lactobacilli has raised questions on its probable adverse effects. Increased organic acids production from the dental plaque is one such adverse effect from a caries point of view (9). Interestingly, it has been expected that lactobacilli can attack the cariogenic micro organisms (10). While the acids produced by other microorganisms in the oral cavity results in the demineralization of tooth probiotics seems to have a contrasting results on oral ecology (11). Therefore, the aim of the present study was to investigate whether the addition of probiotic lactobacilli to dental plaque could affect its acid production.

Lactobacilli are generally microaerophilic or facultative. They are acidogenic, aciduric, extensively spreading and are a part of the normal oral microflora in humans. They normally constitutes < 1% of the total cultivable microbiota in the oral cavity During 20th century, lactobacillus particularly *L. acidophilus* was considered to be the causative agent of caries (12). Through studies it has also been found that L. plantarum can interfere with *S. mutans* (13,14). Thus in the present study we used two strains of lactobacilli namely *L. acidophilus* and *L. plantarum*.

The age group of 7-12 were selected in the study keeping in mind the patient cooperation level and a common presumption that probiotic supplements are more effective in school aged children than in adults (15).

There was an obvious distinction between the lactic acid produced by the suspensions of supra gingival plaque alone and in combination with *L. plantarum* and *L. acidophilus*, more acid being produced by the former. Lactic acid produced by the pure suspensions of both the tested lactobacillus was very less, least being produced by *L. Plantarum*. This finding of our study is in accordance with the in vitro study done by Haukioja A *et al.* (16) which stated that amount of acid produced after fermentation of glucose and sucrose by *L. plantarum* is < 0.35 µg/dl.

Hedberg *et al.* (17) stated a decrease in pH after the fermentation of glucose and sucrose (pH 5.2-6.8) by *L plantarum*, whereas there was a minor increase in the pH after the fermentation of fructose (pH>6.8). Thus, it is possible that the acid produced by probiotic microbes and plaque suspensions is strain dependent and is highly affected by the type of sugar present for fermentation. In between 2 strains of Lactobacilli, *L. acidophilus* produced more acid in comparison with *L. plantarum* in our study which could be because of its increased ability to ferment sugar.

Theoretically, one adverse effect could be that the acid production in the dental plaque rises with a every day administration of probiotic lactobacilli. The production of lactic acid from the lactobacilli strain could be viewed as a double edged sword. The low pH is responsible for the interaction of lactobacilli with other microorganisms and production of antimicrobial substances, while the low pH may influence the demineralization and remineralization procedure as well (18,19). However, the production of acid does not make a bacterial strain cariogenic (16). A study done by Stecksén-Blicks C *et al.* (20) concluded that ong-term consumption of milk supplemented with probiotic lactobacilli reduced caries incidence in preschool children. thus the concern on cariogenesity upon the consumption of lactobacillus can be overlooked. We evaluated only 2 strains i.e. *L. acidophilus* and *L. plantarum* in the present study and the effect of every strain in the oral cavity could be altogether different. Since the study settings between the *in vitro* and *ex vivo* studies are distinctive, comparison of the outcomes is not advocated. Thus further similar *ex vivo* studies need to be done evaluating the acidogenesity and cariogenesity of the remaining commonly used probiotic bacteria.

## Conclusions

Within the limitations of the study, following conclusions can be drawn:

• The lactic acid produced from supragingival plaque was significantly more than that from *L. acidophilus* and *L. plantarum*. However *L. acidophilus* and *L. plantarum*, did not significantly differ in their lactic acid producing ability..

• *L. acidophilus* and *L. plantarum*, both produced significantly more acid when they were combined with supragingival plaque copared to their pure suspensions.
